# Overweight and Obesity Among School Bus Drivers in Rural Arkansas

**DOI:** 10.5888/pcd16.180413

**Published:** 2019-05-16

**Authors:** Karen H. Kim Yeary, Xiaofei Chi, Shelly Lensing, Hannah Baroni, Alesia Ferguson, Joseph Su, Paul A. Estabrooks, Deborah Tate, Laura Linnan

**Affiliations:** 1 University of Arkansas for Medical Sciences, Fay W. Boozman College of Public Health, Little Rock, Arkansas; 2 North Carolina Agricultural and Technical State University, Greensboro, North Carolina; 3 University of Nebraska Medical Center, College of Public Health, Omaha Nebraska; 4 University of North Carolina at Chapel Hill, Gillings School of Public Health, Chapel Hill, North Carolina

## Abstract

**Introduction:**

Obesity is a major public health concern. Compared with other occupational groups, transportation workers, such as school bus drivers, have higher rates of obesity. However, little is known about the body weight and related health behaviors of these drivers, and opportunities for intervention are undetermined.

**Methods:**

We collected multilevel data from school bus drivers working from 4 school bus garages in Little Rock, Arkansas, and their work environment from January through July of 2017. Data on weight, height, sociodemographic characteristics, work factors, weight-related behaviors, and psychosocial variables were collected from 45 drivers. Analyses explored associations between body mass index (BMI; weight in kg/ height in m^2^) and sociodemographic characteristics, work factors, weight-related behaviors, and psychosocial variables. Two focus groups with a total of 20 drivers explored drivers’ perspectives about healthy weight. Observational data at the bus and garage levels were collected through 2 “ride-alongs” and an environmental scan.

**Results:**

Drivers in our sample were predominately overweight or obese (91.1%), and most did not meet dietary or physical activity guidelines. Drivers who were currently dieting had higher BMIs (36.4; standard deviation [SD], 8.2) than drivers who were not dieting (28.5; SD, 7.7); drivers who reported eating less to lose weight had higher BMIs (38.1; SD, 8.5) than those who did not report eating less (29.5; SD, 6.0). Drivers who did not meet physical activity recommendations had higher BMIs (36.5; SD, 9.8) than those who met recommendations (30.9; SD, 4.8). Structural barriers and work stress were significant barriers to achieving a healthy weight. Resources for healthful eating and physical activity were limited in the garage.

**Conclusion:**

Our study provides preliminary data on the prevalence, risk factors, and perceptions of overweight and obesity among school bus drivers. Study data on drivers’ body weight, health-related behaviors, and psychosocial characteristics could serve as a basis for worksite interventions to improve drivers’ health.

**Summary**

**What is already known on this topic?**

Little is known about body weight and related health behaviors of school bus drivers.

**What is added by this report?**

Multilevel quantitative and qualitative data provide information about the environmental context of school bus drivers relevant to dietary behavior, physical activity, and body weight. We also present preliminary data regarding school bus drivers’ body weight–related health behaviors and perceptions.

**What are the implications for public health practice?**

Data from this study can be used to inform a multilevel worksite obesity intervention for school bus drivers.

## Introduction

Rates of obesity are higher among transportation workers, such as school bus drivers, than among other occupational groups (1). Transportation workers are hypothesized to have higher body weight because of the long periods of sedentary activity and shift work and lack of access to healthy eating and physical activity inherent in the job (2). However, little is known about body weight and associated factors specific to school bus drivers.

School bus drivers merit targeted study because their work schedule may differ from other driving professions, resulting in unique barriers or opportunities for healthy weight. Drivers may experience the unique stress of transporting children and interacting with parents (3–5).

Although literature documents the effectiveness of worksite interventions for weight reduction among white-collar workers (6–8), few have targeted transportation workers. Most obesity interventions targeting transportation workers have focused on truck drivers (5,9,10) and city transit workers (4,11). Preliminary research is needed to determine the need and opportunities for intervention specific to school bus drivers. Therefore, we used mixed methods to collect multilevel data from school bus drivers and their work environment to examine: 1) demographic, behavioral, anthropometric, and psychosocial characteristics and to explore associations between these characteristics and body weight; 2) drivers’ perspectives regarding barriers and facilitators to healthy weight; and 3) how the school bus and garage environment may be associated with body weight.

## Methods

From January through July 2017, we studied school bus drivers from 4 garages serving public school districts in Arkansas. All 4 garages participated in survey activities. One garage serving special needs students participated in qualitative and observational activities. All drivers employed in the garages were eligible to participate. Drivers were recruited through flyers posted in the garage and announcements made at monthly garage meetings, and 490 drivers agreed to participate in our survey. Each participant received a $20 gift card. A community–academic partnership was developed that consisted of the transportation director of one participating garage, H.K.K.Y., and drivers. The study was approved by the University of Arkansas institutional review board.

Data from a questionnaire (reporting measured height and weight), focus group reports, observations from school bus “ride-alongs,” and characteristics of the bus garage environment were collected. A convergent design using a case-study framework was used to integrate quantitative and qualitative data by merging the data (12).

### Questionnaire data

Research staff members distributed questionnaires to participating drivers and measured drivers’ height and weight. We assessed sociodemographic and work factors, body weight and related behaviors, health-related perceptions and attitudes, and support for healthy eating, physical activity, and healthy weight (3–5,13,14).

**Sociodemographic and work factors.** Sociodemographic factors assessed were race/ethnicity, sex, date of birth, residence area, education, marital status, family income, and health insurance. We also measured hours worked per week, duration of employment as a school bus driver, and the type of shift worked (3). Use of vending machines at work was also assessed (3). Job strain was assessed through 13 questions that covered 3 job content domains (15). The national mean was used to determine whether drivers had significant job strain (5,16). Supervisory support was assessed through 4 Likert questions (1 = strongly disagree, 2 = disagree, 3 = agree, 4 = strongly agree). The statements included, “My supervisor is concerned about the welfare of those under her/him;” “My supervisor pays attention to what I am saying”; “My supervisor is helpful in getting the job done”; and “My supervisor is successful in getting people to work together.” Items were summed, with higher scores indicating higher supervisory support (15).

**Body weight and related behaviors.** Body weight was measured by using a Tanita BWB-800A scale (Tanita Corporation of America, Inc). Height was measured to the nearest 0.5 cm using a stadiometer. Weight and height was used to compute a continuous measure of body mass index (BMI, weight in kilograms/height in meters squared) (17).

Dietary intake was examined by the Automated Self-Administered 24-Hour Dietary Assessment Tool (18), where 1 weekday and 1 weekend day are assessed. We used the Healthy Eating Index (HEI) components to estimate energy intake and total diet quality (19). Physical activity was assessed by using the adapted International Physical Activity Questionnaire (3,20). Participants were coded as meeting national physical activity recommendations if they reported at least 150 minutes of moderate physical activity per week (21).

Dieting behavior was assessed by 6 factors: 1) the number of pounds participants had to gain before they noticed, 2) the number of pounds participants had to gain before taking action to lose or maintain weight, 3) whether the participant was currently dieting, 4) whether the participant dieted during the past year, and 3) whether the participant was doing anything to lose weight (3,22,23).

Unhealthy and healthy weight control behaviors were assessed by asking participants whether they had engaged in any of 10 dietary behaviors (eg, increasing fruit and vegetables, taking diet pills) (14). Healthy dieting behavior was defined as participants’ engaging in at least 1 healthy weight control behavior and no unhealthy weight control behaviors.

**Health-related perceptions and attitudes.** Perceived work environment related to healthy weight and related behaviors was measured through 5 statements about the work environment: including, “Fruits and vegetables are hard to get at work,” “Physical activity is hard to do at work,” “There is a lot of information at work about healthy eating,” “There is a lot of information at wok about physical activity,” and “There is a lot of information at work about how to have a healthy weight.” Each of the statements had Likert responses: 1 = strongly disagree, 2 = disagree, 3 = neutral, 4 = agree, and 5 = strongly agree (3).

**Support for healthy eating, physical activity, and healthy weight.** These were assessed with a series of 9 questions whereby participants indicated the level of support they received from coworkers, family, and friends to be healthy (3). For example, 1) How supportive are coworkers/friends/family when it comes to having a healthy weight?; 2) How supportive are your coworkers/friends/family when it comes to eating healthy?; 3) How supportive are your coworkers/friends/family when it comes to being physically active? Possible responses were, not at all supportive, somewhat supportive, neutral, moderately supportive, very supportive.

**Importance of healthy eating, physical activity, and health weight for good health.** These were assessed in a series of 3 statements about the participant’s self-rated importance of caloric restriction, physical activity, and having a healthy weight for their own health: 1) Food choices are important for health, 2) Physical activity is important for good health, 3) Having a healthy weight is important for good health. Question responses were strongly disagree, disagree, neutral, agree, strongly agree. (3).

### Focus groups

Researchers trained in qualitative methods conducted 2 focus groups with 10 participants each and collected field notes. The semistructured focus group guide contained open-ended questions to explore the topics of dietary and physical activity behavior situations, attitudes and beliefs surrounding diet and physical activity, and past health program experiences. Sessions were audio-recorded and lasted approximately 1.5 hours. Focus group discussions were transcribed, checked for accuracy, and analyzed with the field notes.

Conventional content analysis was used independently by 2 coders. Codes emerged from key thoughts that recurred in a similar pattern throughout the transcript. Consistent patterns across codes were then identified from which themes emerged (24). Emergent coding and themes were discussed until an agreement was reached (24,25,26).

### Ride-alongs and worksite observations

Research assistants rode with drivers during morning and afternoon shifts to observe driver behaviors. Two ride-alongs were conducted. A structured guide prompted observation of the bus’s physical setting, the driver’s activities, the interaction between the driver and others, conversations on the bus, the observer’s own behavior and responses, and the general experiences of drivers on the route (24). Research staff members collected descriptive and reflective notes (24), with an emphasis on weight-related behaviors (eg, eating during the shift). After the ride-alongs, the research staff used conventional content analysis to analyze their observational data for common codes, which were collapsed into meaningful categories.

Two staff members independently collected environmental data at the garage by using the worksite environment measure (WEM) (13). The social environment was assessed through a brief interview with the transportation director of a participating garage.

### Statistical methods

Descriptive statistics were calculated for all variables and stratified by sex (27). We used *t* tests and χ^2^ tests or Fisher exact tests in univariate analyses to examine differences between sexes, BMI, and obesity (BMI ≥30) with the participant characteristics (sociodemographic characteristics, work factors, weight-related behaviors, and psychosocial variables). Multivariate analyses were not conducted because the preliminary nature of the data precluded meaningful conclusions.

## Results

Drivers in our sample were predominately overweight or obese (91.1%), and most did not meet dietary or physical activity guidelines. Drivers who were currently dieting had higher BMIs (36.4; standard deviation [SD], 8.2) than drivers who were not dieting (28.5; SD, 7.7); drivers who reported eating less to lose weight had higher BMIs (38.1; SD, 8.5) than those who did not report eating less (29.5; SD, 6.0). Drivers who did not meet physical activity recommendations had higher BMIs (36.5; SD, 9.8) than those who met recommendations (30.9; SD, 4.8). Structural barriers and work stress were significant barriers to achieving a healthy weight. Resources for healthful eating and physical activity were limited in the garage.

### Questionnaire

Most drivers were African American; education level ranged from a minimum of a high school diploma or equivalent to college graduate. Most drivers were currently or previously married (Table 1). Slightly more than 50% were women, and most reported an annual household income of $25,000 or more. Mean age of the drivers was 49 years. Most had been school bus drivers for 15 years or less and worked day or split shifts. A little less than half reported working 20 to 29 hours per week. Perceived supervisory support of employees was high. Overall, drivers had limited access to health information related to healthy weight and healthy dietary choices at work. More women than men had household incomes less than $25,000. More men than women had private insurance and more men than women worked 40 hours a week.

**Table 1:** Demographic and Employment Characteristics of School Bus Drivers (N = 45) in Four Garages, Little Rock, Arkansas, June–July, 2017^a^

Characteristic	Total, N = 45	Female, n = 24	Male, n = 21	*P* Value^b^
**Age, mean (standard deviation), y**	48.8 (12.2)	48.2 (13.9)	49.4 (10.3)	.73
**Male**	21	NA	NA	NA
**African American^c^**	40	22	18	.53
**Education**				
High school diploma or general equivalency diploma	18	10	8	.31
Technical or vocational school or some college	19	8	11	
≥College graduate	8	6	2	
**Annual household income, $**				
<20,000	11	9	2	.04
20,000–24,999	9	7	2	
25,000–34,999	6	2	4	
35,000–49,999	10	3	7	
≥50,000	9	3	6	
**Marital status**				
Currently married or member of unmarried couple	17	7	10	.56
Previously married and not currently married	13	8	5	
Never married	15	9	6	
**Health insurance**				
Private	23	9	14	.006
Medicare only	5	4	1	
Medicaid or other public (Veterans Administration, Champus/Tricare)	9	8	1	
None	4	3	1	
Unknown/unreported	4	0	4	
**Years have been a school bus driver**				
0– 6	16	7	9	.63
6–15	14	8	6	
>15	15	9	6	
**Hour per week driving school bus**				
<20	6	2	4	.01
20–29	18	15	3	
30–39	9	3	6	
≥40	12	4	8	
**Usual work schedule**				
Day shift	19	11	8	.77
Split shift	21	11	10	
Irregular or rotating shifts	5	2	3	
**Has supervisory support, mean no. (SD)**	15.9 (2.6)	15.9 (3.1)	15.9 (1.9)	.89
**Job stress, yes**	8	5	3	.70

Abbreviation: NA, not applicable.

a Values are number of bus drivers unless otherwise specified.

b Calculated by *t* test (age, supervisory support), χ^2^, or Fisher’s exact test (categorical measures).

c Other races were white (n = 5) and unknown (n = 1).

More than 90% of drivers were overweight or obese, and most were obese (Table 2). Drivers in our study had low total HEI scores, and a low percentage (32.6%) met CDC national physical activity guidelines. Most drivers reported reducing the amount of food intake in the past year to help lose or to control their weight and practicing healthy weight loss behaviors to lose or control their weight. Only 36% reported weighing themselves at least weekly, and most reported infrequent weighing. Nearly all women in the sample reported doing something to lose weight compared with about two-thirds of men.

**Table 2:** Body Weight and Related Health Behaviors of School Bus Drivers (N = 45) in Four Garages, Little Rock, Arkansas, June and July, 2017^a^

Variable	Total	Female	Male	*P* Value^b^
**Body weight, mean (SD)**	**N = 45**	**N = 24**	**N = 21**	
Weight, kg	100.3 (24.1)	98.2 (21.7)	102.8 (26.9)	.53
BMI^c^, mean (SD)	34.6 (8.7)	35.9 (7.5)	33.2 (9.9)	.29
**BMI^c^, n**				
Underweight	1	0	1	.39
Normal weight	3	1	2	
Overweight	12	5	7	
Obese	29	18	11	
**Physical activity^d^, mean (SD)**	**N = 43**	**N = 23**	**N = 20**	***P* Value** b
Vigorous^e^ (min/wk)	39.5 (89.5)	31.2 (78.1)	49.0 (102.3)	.52
Moderate^e^ (min/ wk)	71.9 (166.7)	64.8 (158.0)	80.1 (180.0)	.77
Walking^e^, min/wk	66.8 (124.3)	47.7 (100.9)	88.8 (146.3)	.29
Sitting^f^, hr/d	5.2 (4.2)	5.2 (3.8)	5.2 (4.8)	.96
Meets physical activity recommendations (150 min/wk)^d^	32.6 (14)	30.4 (7)	35.0 (7)	.75
**Dietary intake, mean (SD)**	**N = 37**	**N = 21**	**N = 16**	***P* Value** b
**Healthy Eating Index–2015 component scores^g^ mean (SD) **				
Kcal/d	1,788.5 (833.1)	1,808.0 (940.3)	1,763.0 (696.9)	.87
Total servings vegetables (0–5)	3.4 (1.6)	3.2 (1.7)	3.7 (1.5)	.34
Greens and beans (0–5)	2.0 (2.3)	1.9 (2.3)	2.1 (2.4)	.76
Total fruit (0–5)	2.1 (2.0)	2.0 (2.0)	2.3 (2.1)	.64
Whole fruit (0–5)	2.0 (2.2)	2.1 (2.3)	1.9 (2.2)	.80
Whole grains (0–5)	2.6 (3.2)	2.4 (3.3)	2.9 (3.3)	.64
Dairy (0–10)	4.4 (3.0)	5.1 (2.9)	3.4 (3.0)	.09
Total protein foods (0–5)	4.7 (0.8)	4.9 (0.4)	4.4 (1.2)	.10
Seafood and plant protein (0–5)	1.9 (2.3)	2.2 (2.3)	1.7 (2.3)	.52
Fatty acid ratio (0–10)	5.9 (3.4)	5.4 (3.7)	6.5 (3.0)	.36
Sodium (0–10)	2.6 (2.8)	2.6 (2.7)	2.5 (3.0)	.89
Refined grains (0–10)	6.6 (3.5)	6.8 (3.1)	6.3 (4.0)	.68
Saturated fat (0–10)	5.3 (3.3)	5.3 (3.3)	5.2 (3.3)	.91
Added sugar (0–10)	6.5 (3.2)	6.2 (3.1)	6.9 (3.4)	.51
Total HEI-2015 Score (0–100)^g^	49.8 (11.9)	50.0 (11.7)	49.6 (12.6)	.94
**Weight control behavior, mean (SD)**	**N = 45**	**N = 24**	**N = 21**	***P* Value^b^**
Currently dieting to lose weight	40.0 (18)	41.7 (10)	38.1 (8)	.83
Not currently dieting	48.9 (22)	50.0 (12)	47.6 (10)	.83
Dieted in past year	26.7 (12)	25.0 (6)	28.6 (6)	.79
Engaged in any weight loss behavior	77.8 (35)	91.7 (22)	61.9 (13)	.03
Reduced food intake	60.0 (27)	70.8 (17)	47.6 (10)	.11
Increased intake of fruits and vegetables	53.3 (24)	54.2 (13)	52.4 (11)	.90
Stopped eating sweets and junk food	55.6 (25)	54.2 (13)	57.1 (12)	.84
Increased exercise levels	35.6 (16)	41.7 (10)	28.6 (6)	.36
Decreased fat intake	35.6 (16)	37.5 (9)	33.3 (7)	.77
Stopped between-meal snacking	35.6 (16)	37.5 (9)	33.3 (7)	.77
Reduced calorie intake	33.3 (15)	33.3 (8)	33.3 (7)	>.99
Skipped meals	22.2 (10)	33.3 (8)	9.5 (2)	.08
Ate no food for at least 24 hrs	8.9 (4)	8.3 (2)	9.5 (2)	>.99
Took diet pills	6.7 (3)	8.3 (2)	4.8 (1)	>.99
Weight loss behavior (healthy)^h^	60.0 (27)	54.2 (13)	66.7 (14)	.39
Weighs weekly	35.6 (16)	29.2 (7)	42.9 (9)	.30
Weighs monthly or every few months	51.1 (23)	50.0 (12)	52.4 (11)	.30
Weighs once a year or less often	13.3 (6)	20.8 (5)	4.8 (1)	.30

Abbreviation: BMI, body mass index; SD, standard deviation.

a Values are number of bus drivers unless otherwise specified.

b Calculated by *t* test (continuous measures), χ^2^ or Fisher’s exact test (categorical measures).

c Calculated as weight in kg divided by height in m^2^. Underweight = <18.5, normal weight = 18.5 to <25, overweight = to <30 obese = ≥30.

d Assessed by using the adapted International Physical Activity Questionnaire (3,19). Participants were coded as meeting national physical activity recommendations if they reported at least 150 minutes of moderate physical activity per week.

e Participants reported days per week and hours and minutes per day during the past 7 days that they spent walking, in moderate and in vigorous physical activities across work, home, and leisure settings. After converting time to minutes, minutes were totaled for the entire week and all settings and averaged to minutes per day.

f Participants reported hr/d, min/d during the weekday and min/d on the weekend separately that they spent sitting in work, home, and leisure settings. After converting time to hours, hours were totaled for the entire week and all settings and averaged to hr/d sitting.

g The HEI-2015 ranged from 0 to 100 with 100 being the maximum score. Higher scores indicate better adherence to the Dietary Guidelines for Americans (28).

h Selected yes for one or more healthy behaviors (reduced food intake, increased intake of fruits and vegetables, stopped eating sweets and junk food, increased exercise levels, decreased fat intake, stopped between-meal snacking, reduced calorie intake) and none of the unhealthy behaviors (skipped meals, ate no food for at least 24 hr, took diet pills) then coded as healthy.

Participants reported higher levels of support from family for healthy food choices, physical activity, and weight management than from their friends (Table 3). Nearly all drivers agreed that healthy food choices, physical activity, and weight management were important for health. Men reported a higher level of coworker support for physical activity than did women.

**Table 3:** Health-Related Perceptions and Attitudes of School Bus Drivers (N = 45) in Four Garages, Little Rock, Arkansas, June and July, 2017

Variable	Total N = 45	Woman n = 24	Man n = 21	*P* Value^a^
**Agree these are hard to get at work, n**				
Fruit and vegetables	30	18	12	.20
Physical activity	17	9	8	.97
**Agree lots of information available at work, n**				
Healthy eating	5	3	2	>.99
Physical activity	5	3	2	>.99
Weight management	5	3	2	>.99
**Receives social support for healthy food choices^b^**				
Family	4.1 (1.3)	4.0 (1.3)	4.1 (1.3)	.71
Friends	3.2 (1.4)	3.4 (1.4)	3.0 (1.3)	.37
Coworkers	2.6 (1.4)	2.6 (1.5)	2.7 (1.5)	.92
**Receives social support for physical activity^b^**				
Family	3.6 (1.4)	3.5 (1.5)	3.8 (1.3)	.46
Friends	3.2 (1.4)	3.1 (1.4)	3.2 (1.3)	.78
Coworkers	2.5 (1.4)	1.9 (1.1)	3.2 (1.4)	.001
**Receives social support for weight management^b^**				
Family	4.0 (1.4)	4.0 (1.5)	4.0 (1.3)	>.99
Friends	3.1 (1.4)	3.1 (1.5)	3.1 (1.4)	.97
Coworkers	2.5 (1.4)	2.3 (1.4)	2.7 (1.4)	.28
**Agrees in general that these are important for health, n**				
Healthy food choices	44	23	21	>.99
Physical activity	45	24	21	>.99
Healthy weight	44	23	21	>.99
**Agrees that these are important for him/her personally, n**				
Eat fewer calories	31	18	13	.34
Physical activity	41	22	19	>.99
Manage weight	45	24	21	>.99
**Weight gain**				
Pounds gained needed to notice, mean (SD)	11.6 (12.8)	9.6 (6.8)	14.0 (17.2)	.28
Pounds gained needed to take action^c^, mean (SD)	18.7 (19.8)	19.5 (21.0)	17.8 (18.6)	.79

Abbreviations: SD, standard deviation.

a Calculated by *t* test (continuous measures), χ^2^ or Fisher’s exact test (categorical measures).

b Calculated as mean and standard deviation of scores, from 1 = not at all supportive to 5 = very supportive.

c School bus drivers were asked how many pounds they would need to gain before they took action to lose weight.

### Univariate analyses

A total of 45 observations were available for analysis. Drivers who were currently dieting had a higher average BMI (36.4; standard deviation [SD], 8.2) than those not dieting (BMI, 28.5; SD, 7.7). Participants who reported eating less to lose weight had a higher mean BMI (38.1; SD, 8.5) than those who had not practiced such behaviors (BMI, 29.5; SD, 6.0). Drivers who did not meet physical activity recommendations also had a higher mean BMI (36.5; SD, 9.8) than those who met recommendations (BMI, 30.9; SD, 4.8).

Dieting behaviors and not meeting national physical activity recommendations were significantly associated with obesity (Figure). Most (80.6%) participants who agreed with the statement, “Eating fewer calories is important for me to be healthy,” were obese, compared with those who did not agree with the statement (28.6%).

**Figure:**
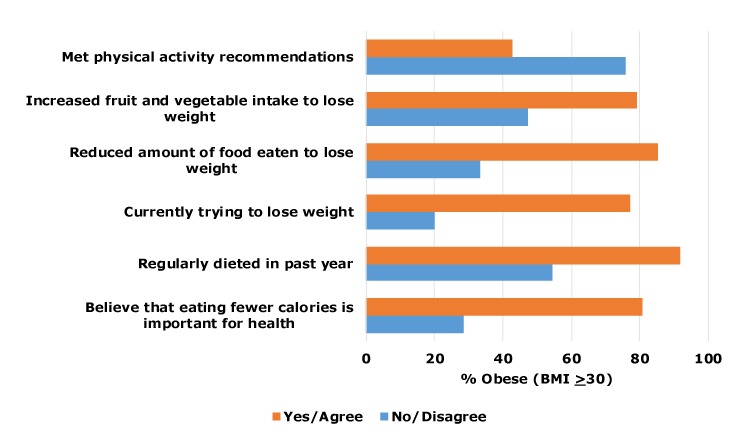
Associations between obesity and dietary, weight-loss, and physical-activity behaviors and beliefs among school bus drivers (N = 45) in 4 Arkansas garages, June and July, 2017. Data are significant at *P* < .05.

**Table:** 

Behavior	No	Yes
Believe that eating fewer calories is important for health	28.6	80.6
Regularly dieted in past year	54.5	91.7
Currently trying to lose weight	20	77.1
Reduced amount of food eaten to lose weight	33.3	85.2
Increased fruit and vegetable intake to lose weight	47.2	79.2
Met physical activity recommendations	75.9	42.9

### Focus groups

Eighty-percent of focus group participants were women, mean age was 56 (SD, 8.6 y), and about half (45%) reported being married or living with a partner. Most participants (70%) reported their education level as having some college or being college graduates or higher. On average, participants were obese (BMI, 32.2; SD, 9.6).

Focus group data yielded several themes that affected drivers’ body weight, including job schedule, work stress, and money. The theme of job schedule captured the consistent pattern of drivers describing their job schedule as a significant barrier to healthy eating, physical activity, and healthy weight. Drivers also spoke at length about the role of myriad forms of work stress in their lives. The theme of money captured drivers’ perceived lack of resources for health, including health insurance and overtime pay. Participant quotes illustrate these points (Table 4).

**Table 4:** Selected Comments, Focus Group Participants (N = 20), School Bus Drivers in 4 Garages in Little Rock, Arkansas, June–July, 2017

Focus Group Theme	Illustrative Quotes
Schedule	“I have a evenin’ run, so usually, I get off by 7:00 pm. I’m not gonna go home and try to cook nothin’. I’m a stop at the fast food restaurant . . . I might eat once a day, if I eat. Then bein’ here [bus garage] sometime, some of us stay here all day.” Woman, aged 47 “All day. We’re gonna snack.” Woman, aged 55 “Right, snack machines, snack, drinks, water. Then we’re gonna go pick up somethin’ to eat for lunch . . . breakfast is almost none and void.” Woman, aged 47 “’Cuz we leave out at 6:00 [AM]. Breakfast don’t kick in till 9:00 AM, 9:30 AM. You don’t have time to stop . . . now, you gotta try to do go somewhere and get some breakfast, or if you go home — me, I’m not driving back across the river cuz that eats up gas, and they don’t pay me enough as it is, so I sit here.” Woman, aged 55 “Getting up this early in the morning to be here at 5:30 AM or 6:00 AM, it’s really nothing open, so when something does open, we swing by there, pick up the quickest thing, and that’s what we throw into our bodies. Then a lot of drivers, they have noon runs. They have [to] be here at 10:00 AM, so some of ‘em don’t even leave because they have to have the runs completed. The first thing they do is run by a fast food place somewhere and grab something.” Woman, age 41 “Working these long hours. The evening run you’re doing at night… When you get home and eat, and I ain’t even lie, sometime I be so tired, I just wanna take a bath and go to bed. Then sometime I go home and I know I’m eating all the wrong stuff.” Man, age 75
Work stress	“Eventually, if you have stress, you have to do somethin’ about it. Some people smoke, some people eat, and some people drink, or some people exercise, but you gotta do something. The higher the stress, the more one of those elements is gonna come into play. Like you said, discipline is not something that is done any more at schools. They [students] can say anything to us, but we can’t say anything to them. It’s hard when you’re an adult when a little child is sittin’ there cursin’ you out and talkin’ about what they gonna do to you.” Man, age 65 “When we’re out there driving, we have to drive for others than ourself, and then we have to deal with the parents. Then they’re calling, complaining. We got to watch the road. We gotta tell the kids to sit down. We got to watch over here, watch over here, and people got it bad. They’ll pass a bus, hop right in front of a bus, and hit brakes. You’re trying to watch kids, and bam! You look up and you’re about to hit this vehicle, so it is [stressful]. Then some of us are single parents, well, like me. I got to focus on my kids, coming to work, make sure that they get home, make sure this, that. Just it’s stressful.” Woman, age 48 “Transportation’s like the stepchildren of any district or private company. We don’t get the respect that a teacher gets. A student can do something to a teacher and get expelled, but if they come on the bus and do it to a driver or an aide, it’s like, ‘Oh well, they have issue.’ Yeah, they have issues, and I understand they have issues, but it’s an issue on how you deal with them. You just can’t say that these drivers and aides don’t matter.” Woman, age 41 “People don’t understand that it really has a toll on your physical… your feet, your knees, your shoulders, your back, and neck.” Woman, age 47
Money	“You’re only scheduled to work 2-and-a-half hours in the morning and 2-and-a-half hours in the evening. If you do anything over . . . just hard about them paying you . . . there’s just excuses. Can’t go to a doctor ‘cuz someone can’t afford the health insurance, like me. I can’t pay no 220-something dollars a month for health insurance through the Marketplace, and I only take home 500-something dollars.” Woman, age 53

Drivers drove the morning (6 am–9 am) and afternoon (2 pm–4 pm) routes each day. Drivers were given the option to add routes, including the noon (10 pm–12 pm), evening (5 pm–7 pm), and field trip routes, for additional compensation. Although some enjoyed the schedule’s flexibility, others said it presented challenges to healthy eating and physical activity. Little time was available for physical activity and meal preparation. Grabbing fast food or already prepared food was more convenient.

Interacting with students and their parents and shouldering the responsibility of transporting students safely caused mental stress. The lack of administrative support to enforce disciplinary action against misbehaving students and parents further exacerbated the stress experienced. Physical stress, such as body aches and pain from the school bus’s bumpy ride, was a deterrent to physical activity.

Drivers stopped receiving health insurance approximately 3 years ago because of policy changes. Drivers also reported not being paid for overtime. Both lack of health insurance and lower salary served as further health barriers.

### Bus ride-alongs

On the 2 ride-alongs conducted, 1 driver was a man aged 72, and the other driver was a woman aged 46. Both routes lasted approximately 2.5 hours and included city streets and interstate highways. Students boarded the bus, sat, and were generally quiet. Drivers picked up each student at their home, greeted some students, and drove students to multiple schools. One driver consumed fast food. Drivers had little downtime during a route, covering areas spread throughout the city in a short amount of time. Traffic ranged from light to moderate. Both drivers altered their routes to accommodate additional stops.

**School bus’s physical setting.** The driver sat up front with a seat belt on a seat that was slightly thicker than the other bus seats. The bus ride was physically uncomfortable, bumpy throughout, and every turn was easily felt. Riders either firmly placed their feet on the ground or held onto the seat to prevent sliding. The busses had fans and lights and cameras above each of the drivers. Both busses had the radio playing a local hip-hop music station. Communication with other drivers and with the dispatcher bus garage was audible on the driver’s radio. There was also a slight rubber or diesel smell.

### Worksite environmental measure

Outside the garage, no resources were available (eg, walking trails) to facilitate physical activity. One large chain grocery store was visible and within walking distance.

One breakroom/common area inside the garage had 1 refrigerator/freezer with an ice machine, 1 water fountain with cups, 1 sink of average condition, 1 microwave, and counter space in good condition. In the front of the break room was a large television in good condition, with 11 tables and chairs arranged to face the television. The television was playing news and soap operas. The breakroom also contained 2 computers in good condition. In the back were 3 vending machines in good condition, containing candy bars and sugar-sweetened beverages. Hot beverages were available from 2 coffee makers. One broken scale was located in the corner of the common area. The garage contained 1 stairwell in average condition and 1 unisex shower. Garages contained no exercise equipment or media about weight management and related health behaviors. The garage director reported limited social support for weight-management.

### Data integration

There was convergence across the data collected. Survey participants reported limited access to health information related to healthy weight and healthy dietary choices at work, which was confirmed by WEM. The lower HEI scores and physical activity levels of the sample were consistent with focus group results reporting limited time for healthy meal preparation and physical activity, ride-along data that reported the physical toll of driving a school bus as a physical activity deterrent, and the WEM showing lack of support for healthy eating and physical activity. The garage director’s report about limited social support for healthy weight within the workplace was also consistent with school bus drivers’ survey results showing less perceived social support from coworkers for healthy behaviors. Focus group participants’ description of the physical stress experienced in driving a school bus was also confirmed by the ride-along data.

In contrast to focus group results that reported the significant role of work stress in deterring healthy behaviors, reported job strain was lower among school bus drivers (18.6%) compared with other transportation groups, such as truck drivers (35.0%) (5). School bus drivers’ perceived stress may be content specific, with drivers reporting lower overall stress, but high stress in the context of healthy behaviors.

## Discussion

Ours is one of the first studies to examine body weight and related behaviors specific to school bus drivers and represents an initial step to understanding a previously underexamined, at-risk population, the environmental context of school bus drivers, and potential avenues for intervention development. The sample was predominately overweight or obese and did not meet dietary or physical activity guidelines, confirming current data regarding transportation workers in general. In univariate analyses, the association between higher engagement in dieting practices and higher BMI was likely due to overweight or obese drivers attempting to lose weight. Drivers who did not meet physical activity recommendations had high BMIs, suggesting the importance of including an emphasis on physical activity in future weight intervention. Work stress was also a prominent theme, with lack of employee health insurance and low pay as distal barriers exacerbating proximal barriers to healthy weight and related behaviors. The WEM also reported limited resources for healthful eating and physical activity. Although this is a convenience sample, the results offer preliminary evidence and a framework for future studies that aim to understand and improve school bus drivers’ body weight.

Future studies with larger, more representative samples may consider examining both distal (eg, socioeconomic status) and proximal (eg, information about healthy eating) factors in examining the role of stress and body weight among drivers. Distal factors may be more prevalent in female drivers given their lower household income, private insurance, and full-time work hours compared with men drivers.

Despite the many challenges to having a healthy weight reported by the drivers in our sample, our study showed feasible opportunities for intervention. Supervisors and drivers represented in our community–academic partnership expressed openness to a worksite weight loss intervention that would increase access to information on healthy behavior related to body weight through current structures within the school bus garage, such as monthly employee meetings. The high levels of coworker support for physical activity among male drivers may be built on to facilitate exercise groups. Education content could include strategies to incorporate physical activity and healthy meal preparation within a driver’s segmented schedule (eg, physical activity in-between driving routes), framing energy reduction as changing eating patterns rather than dieting, and offering stress management techniques (eg, breathing techniques practiced while waiting for children to board the bus). 

Framing healthy eating, physical activity, and healthy weight as important investments of financial and other resources can also be incorporated as an overarching theme in intervention materials, while building on drivers’ current engagement in self-monitoring for weight loss. Given that nearly all of the women drivers reported attempting to lose weight, their interest in weight loss may generate high participation in a worksite weight loss program. Future studies may also benefit from acknowledging structural issues, such as the benefits associated with full-time employment, in intervention development.

The study’s limitations, among others, include its focus on a single workplace that served special needs students for environmental audits and focus groups, which limits external validity. Therefore, we note this significant caveat when making generalizations. Other limitations were the limited external validity of the sample (eg, predominately African American) and not collecting data on whether drivers held other jobs and the household size of SBDs to ascertain the level of poverty within the sample. The cross-sectional nature of the data also precludes conclusions regarding causality. Despite these limitations, the study had numerous strengths. It provided data that can serve as a first step to understanding the body weight, health behaviors, and psychosocial factors of school bus drivers. Our study also provided preliminary understanding regarding the types of intervention approaches that may be successful.
